# Impact of socio-demographic and economic factors on intimate partner violence justification among women in union in Papua New Guinea

**DOI:** 10.1186/s13690-022-00889-0

**Published:** 2022-05-12

**Authors:** Collins Adu, Bernard Yeboah-Asiamah Asare, Williams Agyemang-Duah, Emmanuel Brenyah Adomako, Amma Kyewaa Agyekum, Prince Peprah

**Affiliations:** 1 Department of Health Promotion, Education and Disability Studies, Kwame Nkrumah University of Science and Technology, Kumasi, Ghana; 2 College of Public Health, Medical and Veterinary Sciences, James Cook University, Townsville, Australia; 3 Curtin School of Population Health, Faculty of Health Sciences, Curtin University, Kent Street, Perth, Australia; 4 Institute of Applied of Health Sciences, University of Aberdeen, Aberdeen, Scotland, UK; 5 Department of Geography and Planning, Queen’s University, Kingston, ON K7L 3N6 Canada; 6 Social Work Department, School of Health and Society, University of Wollongong, Wollongong, Australia; 7 Department of Construction Technology and Management, Kwame Nkrumah University of Science and Technology, Kumasi, Ghana; 8 Social Policy Research Centre, University of New South Wales, Sydney, Australia; 9 Centre for Primary Health Care and Equity, University of New South Wales, Sydney, Australia

**Keywords:** Prevalence, Intimate partner violence, Attitude, Economic factors, Cross-sectional studies, Papua New Guinea

## Abstract

**Background:**

Justification of intimate partner violence (IPV) has several implications, including reduced likelihood of help-seeking, increased experiences episodes of partner abuses, and poor health status and outcomes. However, in Papua New Guinea (PNG), where IPV is among the highest globally, little is known about factors influencing IPV justification among women in union. This study aimed at examining the prevalence of IPV justification and associated factors among women in union in PNG.

**Methods:**

Data from the nationally representative cross-sectional demographic and health survey conducted among women aged 15–49 years during 2016–2018 in PNG were used. In all 9,943 women aged 15–49 years who were married or cohabiting during the survey were included. Bivariate and multivariate logistic regressions were performed and the results reported as crude odds ratios (cOR) and adjusted odds ratios (aOR) with 95% confidence intervals (CI).

**Results:**

Overall, almost 7 in 10 women (68.9%, 95%CI:68.0–69.9) justified IPV. Multiple regression analysis revealed that co-habitation (aOR: 1.33, 95%CI: 1.17–1.50, *p* < 0.001), polygyny (aOR: 1.36, 95%CI: 1.20–1.53, *p* < 0.001), exposure to television (aOR: 1.24, 95%CI: 1.08–1.42, *p* = 0.002) and richer wealth status (aOR: 1.19, 95%CI: 1.01–1.40, *p* = 0.035), significantly increased the odds of justifying IPV. We found significantly lower odds of IPV justification among women aged 45–49 years (aOR: 0.53, 95%CI: 0.37–0.77, *p* = 0.001) and those with higher level of education (aOR: 0.56, 95%CI: 0.42–0.74, *p* < 0.001).

**Conclusion:**

The prevalence of IPV justification was high among women in union in PNG. Women’s justification of IPV was associated with socio-demographic and economic factors. Our findings call for appropriate strategies including public education and empowerment programmes that target IPV in PNG. Moreover, strategies and interventions to address IPV justification should target the women’s socio-economic and demographic contexts that influence IPV justification.

## Background

Intimate partner violence (IPV) is a major public health and human right challenge [1, 2] usually leading to long-term physical, biological, psychological and neurological consequences on victims [3, 4]. Physical abuse of women by an intimate partner through battering form 11–30% of physical injuries experienced by women in the USA and Canada, including injuries on their head, neck, thorax, breasts and abdomen [5, 6]. Women who experience IPV are also more likely to experience lethal neurological dysfunctions, cardiovascular challenges, hypertension, reproductive challenges, risk of contracting HIV/AIDS and untimely death [7–9]. A population-based survey of IPV in India in 2014 revealed that 14% of the participants reported severe injuries resulting from IPV [8]. IPV also remains a significant threat to the lives of women globally as well as serving as a barrier to ending women subordination; as part of Sustainable Development Goal (SDG) 5 [10]. Typically, women over the years have been the main victim of IPV across all countries. Almost one-third (27%) of women aged 15–49 years globally experience life-time intimate partner abuse [11]. While its prevalence is overwhelmingly high globally, evidence suggests that the temporal trend is increasing in many developing countries in Asia and Africa [11]. Also, the inter-continental and inter-country level prevalence is increasing significantly, with less than 4% in developed countries to about 75% in some developing countries [11], signaling differential socio-ecological mechanisms reinforcing its continuation in different geographical locations. For example, in a global review of IPV, it was discovered that IPV prevalence could be about 80% in some sub-Saharan African (SSA) countries [12]. Also in North America, about 40–60% mortalities among women are associated with partner violence [5, 8].

Among the Pacific countries, physical, economic, and emotional abuse against women has been increasingly high over the years [3, 7, 13–16]. IPV related women mortality is estimated to vary from 40 to 60% within the Pacific region [8]. Specifically, over 80% of women in Papua New Guinea (PNG) experience IPV, which is believed to be the highest in the world and sometimes leads to serious physical injury [1, 7, 17, 18]. In a study conducted on gender-based violence in PNG [1], it was noted that about 41% of men reported raping their wives within the previous year. When researchers included economic and emotional abuse as IPV forms, prevalence of spousal rape increased to around 87.3% in PNG [14]. Studies report disregard for IPV, particularly intimate partner sexual violence. The disregard for IPV is largely sustained by the notion of privacy^_^ what goes on in private is of no concern to others and the perception that forced sex within marriage or sexual relationships as not rape [19, 20]. Indeed, marital rape has been criminalized in PNG through the passage of the Sexual Offences and Crimes against Children Act 2002 [21]. Again, the PNG constitution states a commitment to equal human rights, and the country has ratified the Convention on the Elimination of All forms of Discrimination against Women. However, IPV is framed within local understandings and perceptions of marriage [19, 20]. IPV, especially physical and sexual violence is considered to be of epidemic rates for many years [19]. The pioneering work of the National Sexual and Reproductive Research Team found that sexual violence that is an everyday occurrence in marriage or partner relationships are ignored in PNG [22]. Kelly-Hanku et al. [23] also reported that IPV especially in the form of sexual violence in the context of marriage is largely unrecognized. Moreover, most cases of IPV in PNG are rarely reported and as a result there is an enduring and dominating silence about women’s experiences of IPV [23]. Given that prevalence of IPV varies largely with geography, it is reasonable to argue that there are complex and strongly nested socio-cultural and economic mechanisms that act to reinforce and perpetuate the act in different socio-spatial settings. From a multilevel standpoint, the micro (the individual), the mezzo (the family), and macro (community) level factors play substantial role in the exposure to IPV [11, 24]. From the individual level, a number of studies  have found personal historical and behavioural factors such as alcohol abuse, younger age, low level of education, childhood exposure to IPV, higher unemployment, and marrying before 18 years as risk factors for IPV [8, 25, 26].

Another important macro-level/contextual determinant of IPV is the community level attitude towards IPV [27, 28]. These contextual factors are deeply entrenched in societal norms, belief systems and mores; which have acted strongly to reinforce gender disparities, that often go against women; sexually, physically, and emotionally [29]. Some researchers have argued that the main macro-level determinant of IPV against women is societal endorsement of power asymmetries and gender stratification that characterize the relationship between men and women [30, 31]. For instance Calvente et al. [32] investigated gender-related ideological and structural macrosocial factors associated with IPV against women in Europe. In that study, it was concluded that ideological gender-related macro factors played a significant role in cross-level interactions with individual-level factors and were thus, good predictors of IPV against women. They discovered that societal attitudes more favourable to gender equality were associated with reduced rates of IPV against women. Conversely a stronger traditional gender role belief and female subordination resulted in higher rates of sexual victimization against women.

It is therefore apparent that, the macro, mezzo, and micro level factors intersect [33, 34] and the outcome of such intersection and interaction shapes how women perceive IPV, and determines whether or not, such abuses are justified [35, 36]. While it is essential that we understand factors that determine women justification of IPV, no study to the best of our knowledge at the time of conducting this study has examined factors that predict women’s justification of IPV in PNG. Although PNG is one of the countries with the highest incidence of IPV, an understanding of the societal and individual factors that influence IPV justification by women has been largely unexplored. This current study therefore seeks to investigate socio-economic and demographic predictors of IPV justification among women in union in PNG based on nationally representative data from the first demographic and health survey conducted in 2016–18. The aim is to provide evidence for targetable policy development, through unravelling the deeply nested socio-demographic and economic factors that explain why women justify IPV in PNG.

## Materials and methods

### Data source, sampling technique and sample size

The study used data from the 2016–18 PNG Demography and Health Survey (PNGDHS) conducted from October 2016 to December 2018. This is the first demographic and survey conducted in PNG. The PNGDHS aimed to generate comprehensive data on demographic, maternal and reproductive issues such as fertility, family planning awareness and practices, breastfeeding practices, health behaviors, immunizations, domestic and intimate partner violence, among others. Through the Demographic and Health Survey (DHS) programme, technical support for the execution of the survey was provided by Inner City Fund (ICF), with the financial support of PNG Government, Australian Government Department of Foreign Affairs and Trade, the United Nations Population Fund (UNFPA) and UNICEF [37]. The sample for the 2016–18 PNG DHS covered the entire population that lived in private dwelling units in the country. The survey used the list of census units (CUs) from the 2011 PNG National Population and Housing Census as the sampling frame and adopted a probability-based sampling approach. Specifically, a two-stage stratified cluster sampling procedure was followed. The methodology and selection procedure details have been reported in the PNGDHS final report.

Each province in the country was stratified into urban and rural areas, yielding 43 sampling strata, except the National Capital District, which has no rural areas. The division paid particular attention to urban–rural variations. Samples of census units were selected independently in each stratum in two stages. In the first stage, sorting the sampling frame within each sampling stratum to achieve implicit stratification and proportional allocation using a probability proportional-to-size selection was done. In the second stage of sampling, a fixed number of 24 households per cluster were selected with an equal probability systematic selection from the newly created household listing, resulting in a total sample size of approximately 19,200 households. To prevent bias, no replacements and no changes of the pre-selected households were allowed in the implementing stages. In cases where a census unit had fewer than 24 households, all households were included in the sample. A total of 17,505 households were selected for the sample, of which 16,754 were occupied. Of the occupied households, 16,021 participated in the study, yielding a response rate of 96%. In the interviewed households, 18,175 women age 15–49 years were identified for individual interviews; interviews were completed with 15,198 women, yielding a response rate of 84%. In this present study, the sample comprised 9,943 women who were in intimate unions during the survey and with complete cases of the variables of interest. We refer to intimate union as marriage or cohabitation. Intimate partners in our study refer to woman who is or was married, in a state registered partnership, or in an intimate or dating relationship with a man presently or at some time in the past. Whereas married in our study refer to being in a state of matrimony, cohabiting refers to a state of living together and having a romantic or sexual relationship without being married on a long-term or permanent basis. Previous related studies in PNG have used same/similar conceptualizations [1, 7, 13].

### Study variables

#### Dependent variable: IPV justification

The dependent variable  of this study is IPV justification. This was obtained from the participants’ responses to the following items: “Sometimes a husband is annoyed or angered by things that his wife does. In your opinion, is a husband justified in hitting or beating his wife in the following situations?” From the PNGDHS and based on previous studies [38, 39], five situations including going out without telling him, neglecting the children, arguing with him, refusing to have sex with him, and burning food were identified. The responses to the questions were coded as ‘yes’ and ‘no’. In this study, women who answered ‘yes’ to at least one of the situations for which a husband hits or beats the wife, were considered as justifying IPV while those who responded ‘no’ to all the five situations were considered as not justifying IPV [9, 36, 38, 40, 41].

#### Explanatory variables; socio-demographic and economic factors

The analysis included several socio-demographic and economic factors that have been theoretically and empirically proven to be significantly associated with IPV justification [9, 35, 38, 40, 41]. Based on previous evidence and their availability in the PNGDHS datasets, we included twelve (12) social, demographic, and economic variables as explanatory variables. These variables included respondent’s age in years (15–19; 20–24; 25–29; 30–34; 35–39; 40–44; 45–49); place of residence (rural; urban); highest educational level (no education; primary; secondary; higher); marital status (married; co-habiting); partner’s age in years (15–24; 25–34; 35–44; 45 + ; 55 +); number of kids (none; 1–2; 3–4; 5–6; 7 and more); husband’s co-wives (yes; no); partner’s educational level (no education; primary; secondary; higher); exposure to television (no; yes); exposure to radio (no; yes); exposure to newspapers/magazines (no; yes); wealth index (poorest, poorer; middle; richer; richest).

### Statistical analysis

Both descriptive (frequencies, percentages, mean and standard deviation) and inferential (chi-square and binary logistic regression) analyses were done using STATA version 13.0 (StataCorp LP, College Station, TX, USA). Descriptive statistics such as frequencies and percentages were presented to describe the demographic and other sample characteristics. The proportion of women who had experienced IPV in the last 12 months and those who justified IPV estimated with confidence intervals. The Pearson’s Chi-square test was done to examine the differences in IPV justification and socio-economic characteristics. Both bivariate and multvariate  logistic regression were performed to model the determinants of women’s justification of IPV. We fitted two regression models to derive both unadjusted and adjusted effects of socio-demographic and economic factors on IPV justification. Model 1 included bivariate regression analysis of all the twelve (12) considered demographic and socio-economic factors. From the results of the bivariate logistic regression analysis, we conducted a multivariate logistic  regression analysis in model 2, where all the statistically significant factors in model one were included. Before the regression analysis, diagnostics checks for multicollinearity were conducted using the variance inflation factor (VIF): result showed a mean VIF of 1.73 (range 1.00–2.82). All the estimates provided in this study were derived by applying appropriate sampling weights supplied by PNGDHS, 2016–18 and the complex survey design to provide unbiased estimates for odds ratio and their confidence intervals. We deleted all missing values from the analysis. The results of the regression analyses were presented as unadjusted odds ratios (cOR) and adjusted odds ratios (aOR) at 95% confidence intervals (CIs). A statistical significance threshold of p ≤ 0.05 was selected.

### Data availability and ethical consideration

The data have been archived in the public repository of DHS. The access to the data requires registration which is granted specifically for legitimate research purposes. Consent forms were administered at household and individual levels, in accordance with the Human Subject Protection. The dataset can be accessed at https://dhsprogram.com/data/ dataset/Papua-New-Guinea_Standard-DHS_2017.cfm?flag = 0.

## Results

### Sample characteristics of the participants

The sample characteristics of the participants by IPV justification are presented in Table 1. The study revealed that 20.4% of the participants were aged 25–29 years, 75.6% resided in urban areas, 49.7% had primary level of education, 99% were Christians and 26.1% rated themselves as richest in terms of wealth status. The study further found that 83% of the participants were married, 18.8% were co-wives, 86.6% were currently living with their partners, 35.3% had 1–2 kids and 37.9% were employed. The results showed that 34.2% of the participants’ partners age ranged between 35–44 years and 43.8% of the participants’ partners had primary level of education. The study found that 24.4% of the participants watched television, 36.5% listened to radio and 37.2% read newspapers/magazines. In a chi-square analysis, the study revealed a statistically significant differences between age groups, place of residence, level of education, wealth index, marital status, co-wives, number of kids, partner’s age, partner’s education level, watching of television, listening to radio and reading of newspapers/magazines in relation to IPV justification among women in union in PNG.

**Table 1:** Distribution of IPV justification across demographic characteristics of women in union

	**IPV Justification**			
**Characteristics**	**Total, n(%)**	***Yes, n(%)***	***No. n(%)***	***p-value***
**Age groups (years)**				< 0.001
15–19	353(3.6)	279(79.0)	74(21.0)	
20–24	1495(15.2)	1059(70.8)	436(29.2)	
25–29	2007(20.4)	1429(71.2)	578(28.8)	
30–34	1896(19.2)	1286(67.8)	610(32.2)	
35–39	1754(17.8)	1201(68.5)	553(31.5)	
40–44	1322(13.4)	866(65.5)	456(34.5)	
45–49	1028(10.4)	674(65.6)	354(34.4)	
**Place of residence**				0.034
Rural	7447(75.6)	5092(68.4)	2355(31.6)	
Urban	2408(24.4)	1702(70.7)	706(29.3)	
**Highest education level**				< 0.001
No education	2262(23.0)	1518(67.1)	744(32.9)	
Primary	4893(49.7)	3435(70.2)	1458(29.8)	
Secondary	2303(23.4)	1613(70.0)	690(23.0)	
Higher	397(4.0)	228(57.4)	169(42.6)	
**Religion**				0.182
Christian	9743(99.0)	6712(68.9)	3031(31.1)	
Non-Christian	52(0.5)	36(69.2)	16(30.8)	
No religion	48(0.5)	39(81.2)	9(18.8)	
**Wealth index**				0.027
Poorest	1481(15.0)	989(66.8)	492(33.2)	
Poorer	1581(16.0)	1085(68.6)	496(31.4)	
Middle	1836(18.6)	1239(67.5)	597(32.5)	
Richer	2385(24.2)	1698(71.2)	687(28.8)	
Richest	2572(26.1)	1783(69.3)	789(30.7)	
**Marital status**				< 0.001
Married	8184(83.0)	5568(68.0)	2616(32.0)	
Co-habitation	1671(17.0)	1226(73.4)	445(26.6)	
**Co-wives**				< 0.001
No	7929(81.2)	5389(68.0)	2540(32.0)	
Yes	1835(18.8)	1350(73.6)	485(26.4)	
**Currently residing with partner**				0.517
Living together	8490(86.6)	5849(68.9)	2641(31.1)	
Staying elsewhere	1317(13.4)	919(69.8)	398(30.2)	
**Number of kids**				0.023
None	1004(10.2)	704(70.1)	300(29.9)	
1–2	3478(35.3)	2450(70.4)	1028(29.6)	
3–4	3195(32.4)	2177(68.1)	1018(31.9)	
5–6	1662(16.9)	1132(68.1)	530(31.9)	
7 and more	516(5.2)	331(64.1)	185(35.9)	
**Occupational status**				0.479
Not working	6035(62.1)	4150(68.8)	1885(31.2)	
Employed	3689(37.9)	2562(69.5)	1127(30.5)	
**Partner’s age (years)**				< 0.001
15–24	711(7.5)	517(72.7)	194(27.3)	
25–34	3204(33.7)	2265(70.7)	939(29.3)	
35–44	3262(34.2)	2234(68.5)	1028(31.5)	
45 +	2340(24.6)	1549(66.2)	791(33.8)	
**Partner’s educational level**				< 0.001
No education	1671(17.4)	1100(65.8)	571(34.2)	
Primary	4207(43.8)	2926(69.6)	1281(30.4)	
Secondary	2914(30.3)	2070(71.0)	844(29.0)	
Higher	812(8.5)	530(65.3)	282(34.7)	
**Watch television**				< 0.001
No	7405(75.6)	5038(68.0)	2367(32.0)	
Yes	2395(24.4)	1725(72.0)	670(28.0)	
**Listen to radio**				0.005
No	6206(63.5)	4213(67.9)	1993(32.1)	
Yes	3570(36.5)	2521(70.6)	1049(29.4)	
**Read newspapers/magazines**				0.009
No	6166(62.8)	4196(68.0)	1970(32.0)	
Yes	3651(37.2)	2576(70.6)	1075(29.4)	

### Prevalence of IPV and IPV Justification

Table 2 presents results on the prevalence of IPV and IPV justification among women in unions in PNG. Overall prevalence of 62.0% IPV in the last 12 months preceding the survey was reported. Prevalence of IPV justification was 68.9% (95%CI = 68.0–69.9) (see Table 2).

**Table 2:** Prevalence of IPV and IPV Justification

Parameter	Percent(95% CI)
IPV	62.0(60.5–63.5)
IPV Justification	68.9(68.0–69.9)

### Predictors of IPV justification among women in union in PNG

Table 3 provides results on the socio-demographic factors influencing IPV justification among women in union in PNG. In the bivariate logistic regression analysis, the study revealed that participants with primary level of education (cOR: 1.15, 95% CI: 1.01–1.23), those living in urban area (cOR: 1.11, 95%CI: 1.01–1.23, *p* = 0.034), those who were co-habiting (cOR: 1.29, 95% CI: 1.15–1.46, *p* < 0.001), those who were co-wives (cOR: 1.31, 95% CI: 1.17–1.47, *p* < 0.001), those whose partners had secondary level of education (cOR: 1.27, 95% CI: 1.12–1.45,*p* < 0.001), those who were exposed to television (cOR: 1.21, 95% CI: 1.09–1.34,*p* < 0.001), radio (cOR:1.14 95% CI: 1.04–1.24,*p* = 0.005) and newspapers/magazines (cOR:1.13 95% CI: 1.03–1.23,*p* = 0.010) and those who rated themselves as richer on wealth index (cOR: 1.23 95% CI: 1.07–1.41,*p* = 0.004) were significantly more likely to indicate IPV as justifiable compared with their counterparts. The study, however, found that participants aged 45–49 years (cOR: 0.50, 95% CI: 0.38–0.67, *p* < 0.001), those whose partners were aged 45 years or above (cOR: 0.73, 95% CI: 0.61–0.88, *p* = 0.001) and those with 7 or more kids (cOR: 0.80, 95% CI: 0.65–0.98, *p* = 0.031) were significantly less likely to justify IPV compared with their counterparts. In the multivariate logistic regression analysis, the study found that participants who were co-habiting (aOR: 1.33, 95%CI: 1.17–1.50, *p* < 0.001), those who were co-wives (aOR: 1.36, 95%CI: 1.20–1.53, *p* < 0.001), those who were exposed to television (aOR: 1.24, 95%CI: 1.08–1.42, *p* = 0.002) and those who rated themselves as richer on wealth index (aOR: 1.19, 95%CI: 1.01–1.40, *p* = 0.035) were significantly more likely to justify IPV compared with their counterparts. The study also revealed that participants aged 45–49 years (aOR: 0.53, 95%CI: 0.37–0.77, *p* = 0.001) and those with tertiary level of education (aOR: 0.56, 95%CI: 0.42–0.74, *p* < 0.001) were significantly less likely to justify IPV compared with counterparts.

**Table 3:** Bivariate and multiple logistic regression of the predictors IPV justification among women in union in PNG

**Predictors**	Unadjusted OR (95%CI)	*p*-value	Adjusted OR (95%CI)	*P*-value
**Age groups (years)**				
15–19	1		1	
20–24	0.64(0.49–0.85)	0.002	0.70(0.51–0.94)	0.019
25–29	0.66(0.50–0.86)	0.003	0.67(0.49–0.92)	0.014
30–34	0.56(0.43–0.74)	< 0.001	0.58(0.41–0.80)	0.001
35–39	0.58(0.44–0.76)	< 0.001	0.61(0.43–0.87)	0.005
40–44	0.50(0.38–0.67)	< 0.001	0.53(0.37–0.76)	0.001
45–49	0.50(0.38–0.67)	< 0.001	0.53(0.37–0.77)	0.001
**Place of residence**				
Rural	1		1	
Urban	1.11(1.01–1.23)	0.034	1.03(0.90–1.18)	0.639
**Highest education level**				
No education	1		1	
Primary	1.15(1.04–1.29)	0.008	1.02(0.89–1.16)	0.815
Secondary	1.15(1.01–1.30)	0.033	0.86(0.73–1.03)	0.094
Higher	0.66(0.53–0.82)	< 0.001	0.56(0.42–0.74)	< 0.001
**Marital status**				
Married	1		1	
Co-habiting	1.29(1.15–1.46)	< 0.001	1.33(1.17–1.50)	< 0.001
**Co-wives**				
No	1		1	
Yes	1.31(1.17–1.47)	< 0.001	1.36(1.20–1.53)	< 0.001
**Partner’s age (years)**				
15–24	1		1	
25–34	0.91(0.75–1.09)	0.282	1.05(0.85–1.30)	0.650
35–44	0.82(0.68–0.98)	0.027	1.00(0.79–1.27)	0.995
45 +	0.73(0.61–0.88)	0.001	0.93(0.71–1.21)	0.584
**Partner’s educational level**				
No education	1		1	
Primary	1.19(1.05–1.34)	0.006	1.13(0.98–1.29)	0.091
Secondary	1.27(1.12–1.45)	< 0.001	1.15(0.98–1.35)	0.083
Higher	0.98(0.82–1.16)	0.784	0.99(0.79–1.24)	0.925
**Number of kids**				
None	1		1	
1–2	0.98(0.84–1.15)	0.851	1.01(0.86–1.20)	0.866
3–4	0.89(0.76–1.05)	0.161	0.98(0.82–1.18)	0.850
5–6	0.90(0.76–1.07)	0.239	1.05(0.86–1.29)	0.601
7 and more	0.80(0.65–0.98)	0.031	1.02(0.80–1.29)	0.892
**Exposure to television**				
No	1		1	
Yes	1.21(1.09–1.34)	< 0.001	1.24(1.08–1.42)	0.002
**Exposure to radio**				
No	1		1	
Yes	1.14(1.04–1.24)	0.005	1.02(0.91–1.15)	0.731
**Exposure newspapers/magazines**				
No	1		1	
Yes	1.13(1.03–1.23)	0.010	1.12(0.99–1.27)	0.077
**Wealth index**				
Poorest	1		1	
Poorer	1.09(0.94–1.27)	0.274	1.09(0.93–1.28)	0.296
Middle	1.03(0.89–1.19)	0.668	1.05(0.89–1.23)	0.564
Richer	1.23(1.07–1.41)	0.004	1.19(1.01–1.40)	0.035
Richest	1.12(0.98–1.29)	0.093	1.06(0.88–1.29)	0.534

## Discussion

This study examined the prevalence and associated predictors of IPV justification among women in union in PNG. The prevalence of IPV justification among women in PNG was 68.9%, comparable to the higher prevalence of IPV justification (76.6%) reported by a previous study in Mali [39]. In contrast, lower rates of IPV justification have been found by previous studies elsewhere in Turkey (15%) [42], Georgia (19%) [43], and Ghana (32%) [44]. It is plausible that the differences in socio-demographic and economic indicators between countries could explain the observed differences in IPV justification.

The current study, like previous findings [40, 42, 45, 46], discovered that socio-demographic factors such as maternal educational status, maternal age group, and marital status were linked to IPV justification among women. IPV justification was associated with maternal educational status, where women with a tertiary degree of education were less likely than women with no formal education to justify IPV. This finding is in line with previous studies in Bangladesh [47], Ghana [40], Malawi [48], Mali [39], and sub-Saharan Africa [49] which found that women with a higher education background were less likely to endorse IPV. Mann and Takyi [50] discovered that uneducated Ghanaian women were more likely to accept violent beliefs. Education is a source of empowerment and a means of achieving independence [51]. Again, education is crucial in influencing women's attitudes against IPV. Educated women may be exposed and well informed about the negatives consequences of IPV and as such see IPV as a negative phenomenon that can harm the victim physically and psychologically [40], whereas less educated women may be less informed about the consequences of such behavior.

Platforms of mass media play a crucial role in social transformation by promoting equality and social inclusion [52]. Inconsistent with the finding of a previous study where women exposed to mass media, were less likely to excuse IPV [46], our current study found women who were exposed to mass media were more likely to justify IPV. Our findings were similar to that of a study in Mali [39], which found that women exposed to mass media were more likely to justify IPV. In our study, women who listen to the radio, read the newspaper, or watch television are more likely to justify IPV. In this context, legislators and government agencies responsible for women's and children's protection should use available media channels to educate the public about the negative consequences of IPV on victims and the societies and to demystify ideations that promote the justification and perpetration of IPV. Given the favorable impacts of exposure to the media on attitudes, stakeholders should use the media to promote awareness about IPV.

Women in the richer wealth index compared to those in the poor wealthindex were more likely to justify IPV, according to the study. This is in line with a previous study in Mali [39], which found that the wealthiest women in Mali were more likely to justify wife violence. In contrast, previous research from Georgia [43] and Ghana [40] found that wealthier women were the least likely to justify IPV. Women's financial independence and less reliance on men for money, could explain the mechanism underlying the link between wealth index and IPV justification among women [40].

Women aged 45–49 were less likely to justify IPV compared to those aged less than 20 years, according to the current study. In Ghana [40, 49], Mali [39], and India [51] elder women were found to be less likely to justify IPV. Women's perceptions and attitudes toward IPV vary as they become older, according to Kathryn and Yount [53] and Waltermaurer et al. [43]. In order to combat oppression and abuse from their personal relationships, older women may develop self-esteem, self-reliance, and self-confidence [39]. Based on our findings,  interventions could focus on helping younger women to develop self-esteem, self-reliance, and self-confidence in order to counteract IPV and its justification among women, with older women functioning as significant others and counseling younger women in home dynamics.

The study revealed that women who reside in urban centers were more likely to see IPV as justifiable compared to their counterparts. However, IPV is more common in rural than urban areas [54]. According to Aboagye et al. [38] living in a rural area is a risk factor for IPV justification among women. The widespread of IPV in PNG [17, 18] could account for the findings in our current study, and more studies may be needed to further explore the justification of IPV among urban women dwellers in PNG. Our result contradicts with previous studies conducted in Kenya [55] and Uganda [56].

There are some limitations in the study that must be noted. The results cannot be used to make a causal conclusion due to the cross-sectional nature. The study also relied on self-reported data, which could be affected by social desirability bias. We recognize that the spread of the variable over the wide response scale would have unleashed all intervening traits of the variable for the analysis to represent to individual differences. Aside from these shortcomings, the study's large sample size and use of a nationally representative dataset may allow the findings to be generalized to PNG women.

## Conclusion

The prevalence of IPV justification was high (68.9%) among women in union in PNG. Justification of IPV was associated with socio-demographic and economic factors including place of residence, educational status, media exposure, marital status, and wealth index. Our findings call for appropriate strategies including public education and empowerment programmes that target IPV in PNG. Moreover, strategies and interventions to address IPV justification should target the women’s socio-economic and demographic contexts that influence IPV justification.

## Data Availability

The datasets used and/or analysed during the current study are available from the corresponding author on reasonable request.

## Abbreviations

IPV: Intimate partner violence
WHO: World Health Organization
SSA: Sub-Saharan Africa
DHS: Demographic and health survey
PNG: Papua New Guinea
ICF: Inner City Fund
